# Corneal Endothelial Cells Provide Evidence of Accelerated Cellular Senescence Associated with HIV Infection: A Case-Control Study

**DOI:** 10.1371/journal.pone.0057422

**Published:** 2013-02-27

**Authors:** Sophia Pathai, Stephen D. Lawn, Paul G. Shiels, Helen A. Weiss, Colin Cook, Robin Wood, Clare E. Gilbert

**Affiliations:** 1 International Centre for Eye Health, Department of Clinical Research, Faculty of Infectious and Tropical Diseases, London School of Hygiene & Tropical Medicine, London, United Kingdom; 2 Desmond Tutu HIV Centre, Institute of Infectious Diseases and Molecular Medicine, Faculty of Health Sciences, University of Cape Town, Cape Town, South Africa; 3 Department of Clinical Research, Faculty of Infectious and Tropical Diseases, London School of Hygiene & Tropical Medicine, London, United Kingdom; 4 Institute of Cancer Sciences, College of Medical, Veterinary & Life Sciences, University of Glasgow, Glasgow, United Kingdom; 5 Medical Research Council Tropical Epidemiology Group, Faculty of Epidemiology and Population Health, London School of Hygiene & Tropical Medicine, London, United Kingdom; 6 Department of Ophthalmology, Faculty of Health Sciences, University of Cape Town, H53 Old Main Building, Groote Schuur Hospital, Cape Town, South Africa; Zhongshan Ophthalmic Center, China

## Abstract

**Background:**

Cellular senescence may be a key factor in HIV-related premature biological aging. We assessed features of the corneal endothelium that are known to be associated with biological aging, and cellular senescence markers in HIV-infected adults.

**Methods:**

Case-control study of 242 HIV-infected adults and 249 matched controls. Using specular microscopy, the corneal endothelium was assessed for features of aging (low endothelial cell density [ECD], high variation in cell size, and low hexagonality index). Data were analysed by multivariable regression. CDKN2A expression (a cell senescence mediator) was measured in peripheral blood leukocytes and 8-hydroxy-2′-deoxyguanosine (8-OHDG; an oxidative DNA damage marker) levels were measured in plasma.

**Results:**

The median age of both groups was 40 years. Among HIV-infected adults, 88% were receiving antiretroviral therapy (ART); their median CD4 count was 468 cells/µL. HIV infection was associated with increased odds of variation in cell size (OR = 1.67; 95% CI: 1.00–2.78, p = 0.04). Among HIV-infected participants, low ECD was independently associated with current CD4 count <200 cells/µL (OR = 2.77; 95%CI: 1.12–6.81, p = 0.03). In participants on ART with undetectable viral load, CDKN2A expression and 8-OHDG levels were higher in those with accelerated aging, as reflected by lower ECD.

**Conclusions:**

The corneal endothelium shows features consistent with HIV-related accelerated senescence, especially among those with poor immune recovery.

## Introduction

Patients receiving antiretroviral therapy (ART) are at increased risk of age-related non-AIDS morbidity and mortality compared with HIV-seronegative persons [1], [2]. It is speculated that as those with HIV age chronologically, they are also likely to undergo accelerated biological aging, mediated by increased cellular senescence. This may be due to replicative senescence (a state of irreversible cellular growth arrest) and stress-induced premature senescence (SIPS) from exposure to environmental stresses, including oxidative stress [3]. Senescent cells adopt an enlarged morphology and secrete inflammatory factors leading to low-level, chronic inflammation described as the senescence-associated secretory phenotype (SASP) [4].

The eye may be a useful site for investigating cellular dynamics of aging, as the corneal endothelium can be readily visualized using non-invasive techniques. The corneal endothelium is a monolayer of mosaic-like cells which lines the inner surface of the cornea. Human corneal endothelial cells (HCEC) do not have substantial replicative potential *in vivo* and their form and quantity influence the health of the cornea [5]. Cells vary from 4 to 6 µm in thickness, are up to 20 µm in width, and have a stable, metabolically efficient hexagonal shape [6]. A key function of the corneal endothelium is to maintain corneal transparency via an ionic ‘pump’. Loss of HCEC beyond a critical threshold results in corneal oedema and loss of visual acuity.

HCEC can be viewed non-invasively via specular microscopy which uses specular reflection [7]. Three endothelium parameters are commonly assessed. Firstly, the number of cells within a defined area - endothelial cell density (ECD), which decreases [8]–[10] throughout life at an average rate of 0.3–0.6%/year [5], [11]. Mean cell density is approximately 3400 cells/mm^2^ at age 15 years declining to approximately 2300 cells/mm^2^ by 85 years [12]. The endothelial response to gradual cell loss is spreading and migration of neighbouring cells, leading to an increase in overall cell size and loss of hexagonal shape [5]. Thus, the second parameter that can be measured is variation in cell size (*polymegathism)* which is- measured using the coefficient of variation (CV) of cell area, which is the ratio between the observed standard deviation and the arithmetic mean of all the cells examined. An average value is less than 35. Variation in cell size increases with increasing chronological age. The final parameter is the hexagonality index – the proportion of cells with 6 sides and this decreases with increasing chronological age [13].

Reduction in proliferative capacity of HCEC is partly mediated by an age-related increase in expression of the cell cycle mediator CDKN2A [14], [15] that functions to hold a cell in a state of growth arrest. Increasing levels of CDKN2A transcriptional expression occur with increasing age, in solid organs and peripheral blood leukocytes [16]–[19]. Reduction in HCEC proliferative capacity may also result from nuclear oxidative damage, which can be assessed by 8-hydroxy-2′-deoxyguanosine (8-OHDG) levels, a physical marker of oxidative DNA damage [3], [20]. If premature cellular senescence occurs in HIV infection, senescent changes in HCEC may be evident in HIV-infected individuals compared to age-matched uninfected controls. Nadir CD4 count, current CD4 count and ART duration have frequently been reported as predictors of increased risk of systemic age-related morbidities in HIV-infected people [21]–[24]. We have previously reported from a South African population that HIV infection is associated with a functional phenotype consistent with frailty [25], and that changes in retinal vessel calibre (as a proxy for systemic vasculature) are consistent with accelerated aging and increased cardiovascular risk [26]. We now report on the cellular aspects of aging within this study cohort, using corneal endothelial cells as readily accessible model of cellular senescence. The objective of this study was to assess differences and identify predictors of HCEC parameters and markers of cellular senescence in a cohort of HIV-infected individuals in comparison to healthy controls.

## Methods

### Study Participants

HIV-infected participants aged 30 years and above were enrolled from a community-based HIV treatment centre in Nyanga district of Cape Town, South Africa [27], [28]. Participant recruitment has been reported in detail elsewhere [25]. In brief, all participants had a confirmed serological diagnosis of HIV and were either about to commence ART (ART-naïve), or were already on first-line ART. A control group of HIV-seronegative participants was recruited using frequency-matching by gender and 5-year age categories.

The study was approved by the London School of Hygiene and Tropical Medicine Ethics Committee and the University of Cape Town Faculty of Health Sciences Ethics Committee, and adhered to the tenets of the Declaration of Helsinki. Written informed consent was obtained from all participants.

### Data Collection

Socio-demographic information and medical history were obtained by questioning participants in their first language (Xhosa or English). Data were also collected on exposures that are known to affect aging and age-related parameters (e.g. smoking, work location as a proxy for UV exposure). Clinical information was obtained from medical case notes where required. All participants underwent a full ophthalmic examination including measurement of visual acuity, evaluation by slit lamp microscopy (for anterior segment structures e.g. cornea, anterior chamber) and indirect ophthalmoscopy (for retina and vitreous).

### Endothelium Assessment

**Figure 1:**
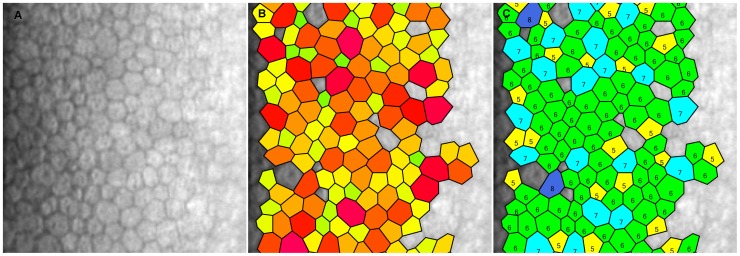
Example of corneal endothelial cells captured by specular microscopy. A: Endothelial cells – density is 2454 cells/mm^2^. B: Polymegathism assessment – measurement of cell size. C: Pleomorphism assessment – proportion of cells that are hexagonal.

A non-contact specular microscope was used (SP02, CSO; Florence, Italy) in automatic release mode to reduce operator-dependent variables. The operator focused and aligned a real-time image of the participant’s eye. The instrument captured the endothelium in the central corneal area, and was calibrated to account for varying thickness of the central cornea (which has the potential to affect endothelial cell counts). The instrument software automatically calculated the quality and reliability of a captured image; if an image was of poor quality (i.e. not ‘ok’ on the image quality specification), the measurement was repeated. Participants who had corneal pathology or evidence of past or current intraocular inflammation were excluded from analysis. Endothelial parameter values were extracted from the image captures in a masked fashion. Two assessments were taken per eye and the mean was used in analyses. Figure 1 shows an example of the output.

### CDKN2A Expression Determination and 8OHDG Measurement

RNA was extracted from peripheral blood leukocytes (PBL) using Trizol reagent (Invitrogen, UK) following manufacturer’s guidelines. RNA extraction was performed in Cape Town and samples shipped on dry ice to the University of Glasgow. Relative quantitative real-time PCR (qRT-PCR) was used to estimate mRNA levels corresponding to the candidate senescence associated gene - CDKN2A. Expression levels were measured against a reference HPRT housekeeping gene on an ABI Prism(R) 7500 Sequence Detection System. Sequences of human TaqMan™ Primer/Probe sets were designed by Primer Express algorithm (Applied Biosystems, Austin, TX, USA). The comparative threshold cycle method (ΔΔCT) was employed to quantify relative gene expression. The quantification result was transformed to an exponential value, 2–ΔΔCt [29] where Ct is the threshold cycle, or the cycle when the product was first detected. Prior to this quantitative study we demonstrated that the efficiency of amplification of reference (HPRT) and test genes were approximately equal (data not shown). Plasma 8-OHDG concentration was quantified using ELISA kits (OxiSelect, Cell Biolabs, CA, USA) according to manufacturer's instructions.

### Statistical Analyses

One eye per person was randomly selected for analyses. Where an eye was not available, for example due to trauma, the contralateral eye was used. Endothelium parameters were assessed as categorical variables by division into quartiles defined in the HIV-seronegative participants. The association of HIV status with these quartiles was assessed in univariable and multivariable logistic regression models adjusted for risk factors and *a priori* confounders (UV exposure, smoking). Linear regression analyses were also performed to assess the rate of endothelial cell loss per year in the two groups using age as a continuous variable. The endothelial parameter phenotypes were further defined with binary categorization to denote ‘aging’ as follows: low endothelial cell count (<25^th^ percentile: <2461cells/mm^2^), high polymegathism (>75^th^ percentile: >39), and low hexagonality index (<25^th^ percentile: <46). Serum biomarkers (CDKN2A and 8-OHDG) were analysed as continuous variables and multiple linear regression models were used to examine the relationships of biomarker expression with endothelial cell parameters. All analyses were performed with Stata 11 IC (Stata Corp, USA).

## Results

### Participant Characteristics

Characteristics of the 242 HIV-infected individuals and 249 uninfected age/gender frequency matched HIV-seronegative individuals are reported in Table 1. HIV-infected participants tended to have a greater income (attributable to a government health-related grant), to be non-smokers, and reported less consumption of alcohol than HIV-seronegative individuals. HIV-infected participants were almost twice as likely to have a history of an eye condition (p = 0.01), but there was little difference in visual acuity (Table 1). None of the participants had undergone intraocular surgery in either eye or had evidence of clinically active uveitis, both of which could have affected the endothelium. Among the HIV-infected participants, 88% were receiving ART, the current median CD4 count was 468 cells/µL (interquartile range: [IQR]: 325–607) and 84% had an undetectable plasma viral load (VL –defined as <50 copies/mL).

**Table 1:** Characteristics of study participants.

Variable	HIV-infected participants (242) % (n)	HIV-seronegative individuals (249) % (n)	P-value
Age (mean±SE)	41.2±0.5	42.5±0.6	0.10
Age, years by group			
30–39	119 (49.2)	117 (47.0)	
40–49	81 (33.5)	79 (31.7)	0.54
>50	42 (17.4)	53 (21.3)	
Male gender	25.6 (62)	24.1 (60)	0.70
Working location			
Indoors	35.1 (85)	28.1 (70)	0.10
Income			
>ZAR1000/month	42.2 (102)	31.7 (79)	0.02
Smoking status			
Non-smoker	85.5 (207)	72.7 (181)	0.001
<10 years	4.6 (11)	12.9 (32)	
>10 years	9.9 (43)	14.5 (36)	
Alcohol			
Yes	30.2 (73)	44.2 (110)	0.001
History of eye condition			
Yes	16.9 (41)	9.3 (23)	0.01
Visual acuity (presenting)	20/25	20/20	0.68
% with visual acuity <20/40	6.2 (15)	10.0 (25)	0.12
*HIV-related characteristics (n = 242)*			
WHO stage			
1/2	27.3 (66)		
3/4	72.7 (176)		
ART* naïve	12.0 (29)		
CD4 count in ART naïve group (n = 29)	170 (84–201)		
Log_10_VL in ART naïve group (n = 19)	4.81 (4.11–5.14)		
Current CD4 count in ART group, µl (n = 213)	468 (325–607)		
Nadir CD4 count in ART group, µl	127 (76–171)		
% with detectable VL in ART group	16.0 (34)		
Peak Log_10_VL in ART group	4.56 (3.85–4.98)		
Duration of ART, months	58 (34–75)		
ART Regimen			
Containing AZT/3TC	59.6 (127)		
Other	40.3 (86)		

* ART =  Anti-retroviral therapy

### Corneal Endothelium Assessment

Three participants had corneal pathology precluding accurate image acquisition (inactive corneal infiltrates of both eyes), leaving 488 eyes for analysis. Endothelium parameter images of sufficient quality were available for 468 (95.9%) eyes. Acquisition of sub-optimal images was related to participant movement and rapid blinking during image capture. Poor quality images were more frequent in participants with a history of an eye condition compared to those without (9.4% vs. 3.3%, p = 0.02) and in those who had HIV infection (5.8% vs. 2.4%, p = 0.06).

### Endothelial Cell Density

Median cell density values were 2660 cells/mm^2^ (IQR: 2433–2851) in HIV-infected participants and 2614 cells/mm^2^ (IQR: 2460–2802) in HIV-seronegative individuals (p = 0.56). The age-adjusted rate of endothelial cell loss was greater in HIV-infected participants compared to HIV-seronegative individuals (0.30% per year vs. 0.15%), although this did not reach statistical significance (p-interaction = 0.16). Table 2 reports the association of the endothelial cell parameters with HIV status. Table 3 reports associations of socio-behavioural and clinical factors with low cell density within the study population. Increasing age was strongly associated with low cell density, both in univariable and multivariable analysis (p-trend = 0.001). In addition to increasing age and male gender, a key finding was that low current CD4 counts were independently associated with low cell density among HIV-infected individuals (Table 4). Participants with CD4 counts <200 cells/µL were almost three times more likely to have a low cell density (OR = 2.77; 95%CI: 1.12–6.81). This association was strengthened when restricted to participants on ART (n = 198) (OR = 4.63; 95%CI: 1.48–14.50– data not shown).

**Table 2:** Association of endothelial cell parameters with HIV status.

Endothelial cell parameters	N (468)	HIV-infected participants (%)	Univariate OR (95%CI)	P-value	Multivariate^§^ OR (95%CI) P-value	
Endothelial cell density (ECD)						
First quartile (lowest)	125	64(28.3)	1		1	
Second quartile	98	38 (16.8)	0.60 (0.35–1.03)	0.18	0.62 (0.35–1.10)	0.22
Third quartile	119	58 (25.7)	0.91 (0.55–1.50)		0.93 (0.55–1.57)	
Fourth quartile (highest)	126	66 (29.2)	1.05 (0.64–1.72)		1.10 (0.65–1.85)	
Polymegathism (variation in cell size)						
First quartile	121	51 (22.5)	1		1	
Second quartile	129	59 (26.1)	1.16 (0.70–1.91)		0.99 (0.58.1.68)	
Third quartile	103	54 (23.9)	1.51 (0.89–2.57)	0.04*	1.52 (0.87–2.67)	0.006*
Fourth quartile	115	62 (27.4)	1.61 (0.96–2.69)		1.99 (1.15–3.45)	
Hexagonality index (cell shape)						
First quartile	142	74 (32.7)	1		1	
Second quartile	117	56 (24.8)	0.84 (0.52–1.36)		0.76 (0.46–1.27)	
Third quartile	109	47 (20.8)	0.70 (0.42–1.15)	0.57	0.59 (0.34–1.00)	0.27
Fourth quartile	100	49 (21.7)	0.88 (0.53–1.47)		0.81 (0.48–1.39)	

* = p-value: test for trend.

** Adjusted for age, gender, UV exposure, BMI, alcohol and cigarette smoking status, any history of an eye condition.

**Table 3:** Association of low endothelial cell density in study population (n = 468).

Variable	Univariate OR (95%CI)	P-value	Multivariate§ OR (95%CI)	P-value
HIV infection				
No	1		1	
Yes	1.17 (0.78–1.77)	0.45	1.14 (0.74–1.76)	0.56
Gender				
Male	1		1	
Female	0.74 (0.47–1.18)	0.20	0.66 (0.39–1.14)	0.14
Age (years)				
30–39	1		1	
40–49	1.60 (0.99–2.57)		1.60 (0.98–2.61)	
>50	2.41 (1.41–4.15)	0.005*	2.43 (1.39–4.24)	0.001*
Work location				
Outdoor worker	0.96 (0.62–1.49)		0.87 (0.55–1.37)	
Indoor worker	1	0.85	1	0.55
Cigarette smoker				
No	1		1	
Yes	0.87 (0.52–1.46)	0.60	0.66 (0.34–1.24)	0.20
Alcohol consumption				
No	1		1	
Yes	0.89 (0.58–1.36)	0.58	0.83 (0.51–1.36)	0.46
History of eye condition				
No	1		1	
Yes	1.71 (0.96–3.06)	0.07	1.41 (0.77–2.57)	0.27

* = test for trend.

§ Adjusted for all other variables in model.

**Table 4:** Multivariable models to demonstrate association of endothelial cell parameters in HIV-infected individuals (n = 217).

Variable	Low endothelial cell density§		High polymegathism§ (cell variation)		Low hexagonality index§ (cell shape not 6-sided)		
	OR	P-value	OR	P-value			
Gender							
Male	1		1		1		
Female	0.43 (0.19–0.91)	0.04	2.19 (0.81–5.86)	0.12	0.99 (0.44–2.29)		0.98
Age (years)							
30–39	1		1		1		
40–49	2.84 (1.35–5.97)		1.00 (0.43–2.31)		0.63 (0.30–1.34)		
>50	5.42 (2.21–13.26)	<0.0001*	2.44 (0.97–6.13)	0.09*	1.69 (0.71–3.98)		0.12
Work location							
Outdoor worker	0.57 (0.29–1.12)		2.20 (1.00–4.90)		1.97 (1.00–3.95)		
Indoor worker	1	0.11	1	0.05	1		0.05
Cigarette smoker							
No	1		1		1		
Yes	1.04 (0.39–2.78)	0.93	3.41 (1.13–10.31)	0.03	1.09 (0.39–3.06)		0.87
History of eye condition							
No	1		1		1		
Yes	1.04 (0.44–2.49)	0.93	0.95 (0.37–2.49)	0.93	0.18 (0.05–0.62)		0.01
WHO clinical stage							
1/2	1		1		1		
3/4	1.58 (0.71–3.51)	0.26	1.88 (0.80–4.54)	0.16	1.11 (0.52–2.33)		0.79
Peak viral load							
<10,000 copies	1		1		1		
>10,000 copies	0.65 (0.32–1.31)	0.23	0.87 (0.40–1.86)	0.71	0.84 (0.42–1.69)		0.62
Current CD4 count							
<200 cells/µl	2.77 (1.12–6.81)		1.30 (0.49–3.48)		0.62 (0.24–1.61)		
>200 cells/µl	1	0.03	1	0.60	1		0.33

§ Adjusted for all other variables in model.

* = test for trend.

### Endothelial Cell Polymegathism (Variation in Cell Size)

The median variation in cell size (polymegathism) was 36 (IQR: 33–39) in HIV-infected participants and 35 ([IQR: 32–38], p = 0.07) in HIV-seronegative individuals. HIV was associated with higher polymegathism quartiles in univariable analyses (p-trend = 0.04), and this association strengthened after adjustment (p-trend = 0.006) (Table 2). HIV infection was also associated with high polymegathism as a binary variable (OR = 1.67, 95%CI 1.00–2.78) (Table 5). Increasing age, an outdoor work location and increased duration of smoking were also associated with high polymegathism. However, despite the association of HIV infection with this variable, HIV-related covariates were not related. Independent predictors in HIV-infected individuals were outdoor work (OR = 2.20; 95%CI: 1.00–4.90) and cigarette smoking (OR = 3.41; 95%CI: 1.13–10.31) (Table 4).

**Table 5:** Association of high polymegathism (increased variation of cell size) in study population (n = 468).

Variable	Univariate OR (95%CI)	P-value		Multivariate§ OR (95%CI)	P-value
HIV infection					
No	1			1	
Yes	1.26 (0.78–2.01)		0.34	1.67 (1.00–2.78)	0.04
Gender					
Male	1			1	
Female	1.54 (0.86–2.78)		0.15	2.79 (1.35–5.77)	0.006
Age (years)					
30–39	1			1	
40–49	1.43 (0.83–2.48)			1.31 (0.74–2.33)	
>50	2.33 (1.28–4.25)		0.02	2.33 (1.23–4.40)	0.01*
Work location					
Outdoor worker	1.78 (1.02–3.10)			1.76 (.100–3.12)	
Indoor worker	1		0.04	1	0.05
Cigarette smoker					
Nil	1			1	
5 yrs or less	1.91 (0.72–5.07)			2.07 (0.71–6.02)	
6–15	1.27 (0.46–3.16)			2.34 (0.73–7.44)	
16–20	1.69 (0.65–4.44)			2.47 (0.83–7.39)	
>20	1.87 (0.75–4.64)		0.43	2.72 (0.91–8.12)	0.02*
Alcohol consumption					
No	1			1	
Yes	0.77 (0.47–1.22)		0.26	0.70 (0.39–1.24)	0.22
History of eye condition					
No	1			1	
Yes	1.71 (0.96–3.06)		0.07	0.80 (0.38–1.72)	0.57

* = test for trend.

§ Adjusted for all other variables in µodel.

### Hexagonality Index (Cell Shape)

The median hexagonality index was similar in both groups (50 [IQR: 45–54] vs. 50 [IQR: 46–54], p = 0.55). Low hexagonality index was associated with increasing age (p-trend = 0.006) and outdoor work location (OR = 1.70; 95%CI 1.04–2.78) but there was little evidence of an associated with HIV infection (OR = 1.40, 95%CI 0.90–2.17). In HIV-infected individuals, low hexagonality index was associated with outdoor work (OR = 1.97; 95%CI: 1.00–3.95); and inversely related with a history of an eye condition (OR = 0.18; 95%CI: 0.07–0.62) (Table 4). No HIV-related covariates were associated with this endothelial parameter.

### Association between Markers of Cellular Senescence and Endothelial Cell Density

CDKN2A measurements were available for 430 participants, (221 HIV+/209 HIV−). CDKN2A transcriptional expression was higher in HIV-infected participants compared to HIV-seronegative individuals (0.46 vs. 0.37, p = 0.007). In ART-treated participants with suppressed viral load (<50 copies/ml; n = 155) adjusted CDKN2A expression was higher in those with low cell density compared to those with high cell density (0.57 vs. 0.43, p = 0.04). There was no evidence to suggest an association between CDKN2A expression and cell density in HIV-seronegative individuals (p = 0.33).

8-hydroxy-2′-deoxyguanosine (8-OHDG) levels were available for 70 individuals. 8-OHDG levels were positively associated with CDKN2A expression in HIV-seronegative individuals, however the association was non-significant (r = 0.13, p = 0.33). Mean levels were 0.21ng/ml in HIV-seronegative individuals and 0.22 ng/ml in HIV-infected individuals (p = 0.83). In participants on ART with undetectable viral load (n = 28), those with low ECD count had higher 8-OHDG levels compared to those with high cell density (0.25 ng/ml vs. 0.19 ng/ml, p = 0.04). There was no association between 8-OHDG levels and cell density in HIV-seronegative individuals.

## Discussion

We have already demonstrated in this South African study population that HIV infection is associated with an increased risk of frailty, providing evidence that this functional phenotype is associated with HIV-related accelerated aging [25]. We have also investigated for evidence of aging at an organ/systems level using the retinal vessels as a model of the systemic vasculature, and our findings suggested that HIV infection is associated with changes in the retinal vasculature that may reflect premature aging and increased cardiovascular risk [26]. These previous studies both provided important evidence that HIV infection is associated with accelerated senescence. The present study further builds on these findings, using corneal endothelial cells to represent a readily accessible cellular model of senescence. Here we have demonstrated that HIV-infected individuals have an increased risk of greater corneal endothelial cell size variation (polymegathism) when compared with HIV-seronegative individuals. Furthermore, HIV-infected individuals with a current CD4 count <200 cells/µL were more likely to have low endothelial cell densities than those with higher CD4 counts. These data were corroborated by markers of cellular damage and senescence which were found to be higher in virally suppressed ART-treated individuals with low cell densities. These findings provide further evidence of accelerated aging in HIV, and that this may be influenced by the level of immunodeficiency, as reflected by CD4 count.

Independent predictors of low cell density in HIV-infected individuals included age (as anticipated), male gender and low current CD4 count. The relationship with male gender is likely to be related to the anti-oxidant and anti-inflammatory properties of oestrogen [20], while the relationship with low current CD4 count suggests that poor immune restoration may accelerate senescence. Our finding that UV exposure and smoking were also independent predictors of polymegathism in the overall study population and in HIV-infected individuals supports the findings of others that stress, including oxidative stress, is likely to be an important contributor to cellular senescence mechanisms in HCECs [30]. Our observation of increased CDKN2A levels in PBLs from HIV-infected individuals compared with HIV-seronegative individuals supports the mechanism of systemic oxidative stress. Furthermore, exposure to cigarette smoke has been demonstrated to degrade the function of the cornea through generation of reactive oxygen species [31]. It is surprising that polymegathism was not associated with current CD4 count or other HIV-related covariates in our study. This may suggest that ‘stress’ induced by HIV-related chronic inflammation may lead to HCEC senescence, the initial features of which are an enlarged (or variation in) morphology. Severe immunodeficiency (manifest as a low CD4 count) may be associated with later features of the senescence pathway, ultimately leading to cell cycle arrest and cell loss.

Despite the association of greater polymegathism (variation in cell size) with HIV, we did not detect a difference in cell density between the two groups. The endothelial response to gradual cell loss is spreading and migration of neighbouring cells with an increase in overall cell size and adoption of a non-hexagonal shape [5]. Therefore an overall decrease in ECD might be have been expected. However, as increased polymegathism reflects a *variation* in cell size (not simply an increase in cell size), similar ECD between the two groups is not an unusual finding: ECD can remain the same with different levels of polymegathism. Other studies have reported similar findings [32] and patients with diabetes develop increased polymegathism while retaining normal endothelial cell density for their age [33]. Stress to the endothelium can also lead to cell border changes, with cells expressing a large anterior surface area with a small posterior surface area or vice versa [34]. Capturing a two-dimensional image with the specular microscope may, therefore, provide unchanged cell density measures despite greater polymegathism.An aggregate of phenotypes has been described for senescent cells [4], [35], including irreversible growth arrest, an enlarged morphology, expression of CDKN2A and the senescence-associated secretory phenotype (SASP). This cell phenotype is associated with secretion of cytokines, growth factors and proteases that can lead to low-level chronic inflammation that is a feature of normal aging, and also a key feature in HIV infection [36]. In our study, higher CDKN2A expression in peripheral blood leukocytes and 8-OHDG levels plasma were observed in participants on ART with undetectable viral load and low endothelial cell density, suggesting that accelerated cellular senescence may be occurring systemically. Markers of cellular senescence have also been detected within endothelial corneal tissue, from human subjects and mouse senescence models, showing greater CDKN2A expression with increasing age [37], [38]. In addition, HCECs exhibit signs of oxidative DNA damage, and significantly higher levels of 8-OHDG have been reported in corneas from older donors compared with younger donors [3]. Our findings suggest that corneal endothelial cells in HIV-infected individuals exhibit some of the phenotypic features of senescent cells. Consequently, they would be expected to express more CDKN2A and have the associated SASP compared to HIV-seronegative individuals. This phenotype may also provide a possible mechanism to account for the substantial excess risk of conjunctival malignancies (particularly squamous cell carcinomas [SCC]) seen among HIV-infected individuals [39]. An underlying infectious aetiology for these malignancies has been investigated, but none found to date [40]. It is possible that SASP-associated chronic inflammation could lead to metaplastic and/or neoplastic change within conjunctiva as well as contributing to accelerated cellular senescence. Measurement of CDKN2A expression in conjunctival SCC specimens from HIV-infected individuals would be the ideal next step to investigate this hypothesis.

A key strength of our study is the inclusion of an age- and gender-matched control group with a similar socio-demographic profile to the HIV-infected participants. The hypothesis of accelerated aging in HIV has received criticism due to limitations in characterization of participants, in particular the possibility of differential exposure to potential risk factors between HIV-infected and uninfected populations [24], [41], [42]. By recruiting from the same community, we reduced the likelihood of differential risk exposure. HCEC change morphology and assume an ‘aged phenotype’ in several chronic systemic diseases (e.g. renal failure and diabetes) [43] [44], [45], suggesting that they may be useful in assessing cellular senescence, particularly as measurement is objective and non-invasive.

There are some limitations to our study. Although our hypothesis relates to accelerated aging in HIV, it is possible that subclinical intraocular inflammation could account for the observed relationship between lower CD4 counts and low endothelial cell density. HIV can replicate within the eye causing uveitis [46], and a low CD4 count is a risk factor for immune recovery uveitis (IRU) [47] which could arise in patients with a history of cytomegalovirus (CMV) infection and could potentially cause subclinical endothelial damage. However, we did not detect any evidence of IRU, and we have previously shown the prevalence of CMV retinitis to be very low within this population [48]. Therefore, our data support the hypothesis of premature aging in line with systemic co-morbidities [21], [49]. Another limitation is that misclassification of smoking and alcohol consumption status may have occurred, with HIV-infected participants wanting to demonstrate ‘healthy behaviour’, which could have led to confounding. Our measure of location of work as a proxy measure of ultra-violet exposure may also have been confounded by socio-economic status. Finally, CDKN2A expression and 8-OHDG levels were determined in peripheral blood leukocytes and plasma, which may not directly reflect levels in corneal tissue therefore some caution should be excised when interpreting these differences. However, a number of studies indicate that CDKN2A transcriptional expression increases with increasing chronological age in both leukocytes and solid organs [16], [17], [19]. A direct comparison of CDKN2A and 8-OHDG levels we believe is novel in this context. The positive correlation (although not significant) is intuitive, with more DNA damage reflected in more senescent cells, as determined by CDKN2A expression levels. Our study may simply be limited by lack of power in the 8-OHDG samples measured.

In summary, corneal endothelial cells demonstrate features of senescence in HIV-infected individuals, suggesting HIV infection contributes towards accelerated cellular senescence. Evaluation of corneal endothelial cells in longitudinal studies of HIV-related accelerated biological aging may reveal further insights into the mechanisms of cellular senescence in this population.
